# Dental Ethics

**Published:** 1898-07

**Authors:** S. H. Guilford


ARTICLE VII.

### DENTAL ETHICS.

PROF. S. H. GUILFORD.

Ethics is defined as "the science of right conduct and character," and again as "the doctrine of man's duty in respect to himself and the rights of others." More specifically it is designated as "rules of practice in respect to a single class of human actions and duties." In this latter sense it is used under the term "Dental Ethics."

Individuals are governed as to their conduct in two ways: one, by the enacted law of the nation, state or community, which compels them to do certain things and not to do certain other things; the other, by what is known as the moral law, which regulates their actions in matters not covered or provided for by the enacted law. Under the enacted law man has no choice. He must do what it says or incur a penalty. Under the moral law, which is founded upon the Golden Rule of "doing unto others as we would be done by," we are free to do as we will, either to respect the natural and inherent rights of others or to disregard them. If by our actions and conduct we have regard for the dues of others, we merit and obtain the esteem of our fellow-men and the approval of our conscience; while if we overlook or neglect them, we correspondingly suffer in the opinion of the world at large.

Our actions as practitioners of dental science are regulated by common law only in respect to a general or more important point. It requires of us that we shall be possessed of average skill in the performance of our operations; that we shall exercise reasonable care to prevent accident and misfortune; that we shall inflict no injury and practice no extortion.

It does not, however, provide against misleading the public by false claims of skill, by specious advertising, by doing unsatisfactory work, by condemning the work of fellow-practitioners, or by deceiving or taking advantage of our patients in a variety of ways.

To remedy these defects in our common law each profession has, by general consent, established an unwritten law, commonly known as the code of ethics; which provides in a general way that unless each practitioner pursues his vocation in accordance with just principles he shall be debarred from certain esteemed privileges and forfeit the respect of his fellow-practitioners.

There exists to-day a code of dental ethics formulated and promulgated by the former American Dental Association, a body in the greatest degree representative of the best talent and highest professional standing in our calling. This code, though lacking completeness in many respects, is accepted as embodying the principles of right practice, and has been adopted as a qualification for membership in nearly all the subordinate dental societies in our land.

The first article treats of "The Duties of the Profession to Their Patients."

It declares that the dentist should fully recognize the obligations involved in the discharge of his duties toward his patients, that his manner should be firm, yet kind and sympathetic, and that the simplest case committed to his care should receive the same attention that would be bestowed upon a more important one. It also advocates instructing the public by conversation and explanation while operating for them, so that they may understand, at least in a general way, what we are doing for them and why, so that they may properly and intelligently apyreciate the character and importance of our services.

Furthermore, it enjoins the dentist to be scrupulously clean, both as to his person and surroundings and the instruments and appliances that he employs.

It advises the dentist to be temperate in all things, keeping both mind and body in the best possible condition, so that by skill and clearness of judgment he may be able to confer upon his patient the benefit which it is his right to expect and receive.

In article second it deals with the "Maintaining of Professional Character."

It asserts that a member of the dental profession is bound to maintain its honor and to labor earnestly to extend its sphere of usefulness, that he is to avoid everything in language and conduct that would be calculated to dishonor his calling, and that he should manifest a due respect for his brethren.

By his dress, his office arrangements, his manner and conversation he should give full evidence of being a gentleman and in private or public life show forth a high moral character.

It denounces as unprofessional all public advertising, as by cards, hand-bills, posters or signs, calling attention to peculiar styles of work, prices for services, special modes of operating, or claiming superiority over neighboring practitioners. Also the selling or recommending of nostrums, the solicitation of work or guaranteeing the durability of operations. This article, however, does not discountenance the insertion of a plain business card in newspapers, the issuing to his own patients of special notices of removal, absence from or return to business, or the use of appointment cards having a fee-bill attached.

It declares that when a dentist is consulted by the patient of another practitioner he shall serve him faithfully, and not by word, look or insinuation do anything to weaken the patient's confidence in his own dentist. Furthermore, if the case will not suffer thereby, only temporary work should be done and the patient be referred back to his family dentist.

If the former dentist has been faithful and reasonably skillful, no inducement should he held out to have the patient become one of your clientele.

If a schedule of fees has been arranged by the dentists of any locality, it is highly dishonorable for any individual to violate the agreement in any particular.

In case of laboring people, shop-girls, servants or ministers, whose income is small, however, it is only just and proper that they should receive a reduction from the usual fees.

Article third treats of "The Relative Duties of Dentists and Physicians," and declares it should ever be borne in mind that dental surgery is a specialty in medical science, and that as the de tist is limited to operations in the oral cavity it naturaily follows that he must be more familiar with diseases in this region, and all such cases should be referred to him by the medical practitioner. At the same time, with diseases of the general system the physician is supposed to be more familiar, and the dentist should not transcend his sphere in attempting to treat them, but should refer them to the medical practitioner or proper specialist.

The last article considers "The Mutual Duties of the Profession and Public." It declares that as the dentist is the best judge of the impositions practiced by empirics and quacks, it is his duty to warn the public in regard to them and their harmful methods and practices, and that in doing so and for the benefit he is thus conferring he should receive in return the full confidence of the public.

Finally, it asserts that the dentist should be fully compensated for any and all services whether rendered in the form of actual operations or of professional advice, and that when he is called upon to make an examination or give an opinion in a certain case, he is just as much entitled to remuneration for such service, as though he had devoted the same time and skill to a technical operation.

The subject of ethics, while worthy of consideration at all times by all practitioners, is especially commended to the large number of dental graduates yearly issuing from our colleges. If they are imbued with its principles and start out in practice along the lines it indicates, their careers will be both honorable and successful.—*The Stomatologist.*

